# Clinical Outcomes of Patients With B-Cell Non-Hodgkin Lymphoma in Real-World Settings: Findings From the Hemato-Oncology Latin America Observational Registry Study

**DOI:** 10.1200/GO.21.00265

**Published:** 2022-04-29

**Authors:** Miguel Pavlovsky, Daniel Cubero, Gladys Patricia Agreda-Vásquez, Alicia Enrico, Maria J. Mela-Osorio, Jorge Armenta San Sebastián, Laura Fogliatto, Roberto Ovilla, Oscar Avendano, Gerardo Machnicki, Paula Barreyro, Damila Trufelli, Pamella Villanova

**Affiliations:** ^1^ Servicio de Hematología e Investigación Clínica, Fundación para Combatir la Leucemia (FUNDALEU), Buenos Aires, Argentina; ^2^ CEPHO/ABC School of Medicine, Santo André, Brazil; ^3^ Instituto Nacional de Ciencias Medicas y Nutricion Salvador Zubirán, Mexico City, Mexico; ^4^ Hospital Italiano La Plata, Buenos Aires, Argentina; ^5^ Centro Oncológico Estatal ISSEMYM, Toluca, Mexico; ^6^ Hospital de Clínicas de Porto Alegre, Brazil; ^7^ Hospital Angeles Lomas, Huixquilucan, Mexico; ^8^ Medical Solutions S.A. Guatemala City, Guatemala; ^9^ Janssen-Cilag Farmacêutica Ltda, Buenos Aires, Argentina; ^10^ Janssen-Cilag Farmacêutica Ltda, São Paulo, Brazil

## Abstract

Real-world evidence on non-Hodgkin lymphoma (NHL) management in Latin America is currently lacking. The objective of this study was to describe treatment characteristics and outcomes of NHL in Latin America.

**METHODS:**

A total of 2,967 patients with NHL with aggressive and indolent subtypes, including diffuse large B-cell lymphoma (DLBCL), follicular lymphoma (FL), mantle-cell lymphoma (MCL), and mucosa-associated lymphoid tissue (MALT) lymphoma, with incident or prevalent diagnosis between 2006 and 2015, were retrospectively identified using clinical charts registered in the Hemato-Oncology Latin America Observational Registry. Associations between treatment regimen and age at diagnosis with clinical outcomes within each subtype were estimated using Cox proportional hazard regression.

**RESULTS:**

Most patients with NHL received 1L chemoimmunotherapy, most commonly cyclophosphamide, doxorubicin, vincristine, and prednisone (CHOP) with/without rituximab. Five-year survival rates were higher for MALT lymphoma (90.8%) and FL (87.6%) versus DLBCL (69.0%) and MCL (57.1%), with variations between countries. The median overall survival from first relapse for patients with DLBCL was 6.6 years, with lower risk of death for those diagnosed at age < 65 years (hazard ratio = 0.732; *P* = .0161). Patients achieved a longer median progression-free survival with 1L rituximab-CHOP (R-CHOP) versus CHOP or rituximab, cyclophosphamide, vincristine, and prednisone (RCVP) (7.7 *v* 3.0 or 1.8 years, respectively). Use of regimens other than R-CHOP was associated with a higher risk of death/progression for patients with DLBCL (rituximab, ifosfamide, carboplatin, and etoposide/ifosfamide, carboplatin, and etoposide) and FL (CHOP). There was no relationship between treatment prescribed and age at diagnosis with outcomes from first/second relapse in DLBCL and FL.

**CONCLUSION:**

Differences in treatment outcomes between NHL subtypes were observed, reflecting variations in NHL management and barriers to treatment access in Latin America. These data provide necessary evidence to understand NHL management in this region and highlight the need to improve treatment outcomes for these patients.

## INTRODUCTION

Non-Hodgkin lymphoma (NHL) is a heterogeneous group of hematologic malignancies resulting from uncontrolled proliferation of lymphoid tissues.^1^ NHL has diverse genotypes, immunologic phenotypes, molecular biology, morphology, and clinical characteristics. Central and South America shoulder 7% of the world's burden of NHL, including 27,000 incident cases and 14,000 deaths each year.^2^ Based on these data, NHL is the 9th-ranked cancer diagnosis and the 11th-ranked cause of cancer death in Latin America. By 2030, numbers of NHL incidence and deaths are projected to increase by 60% (to 43,000 cases and 24,000 deaths) in the region.^2^

**CONTEXT**

**Key Objective**
There is a lack of evidence on real-world outcomes of diagnosis, staging, and treatment of patients with non-Hodgkin lymphoma (NHL) in Latin America. The aim of this study was to describe the treatment characteristics and clinical outcomes of patients diagnosed with NHL who received systemic therapy in this region.
**Knowledge Generated**
Nearly 3,000 patients with NHL from seven Latin American countries were included and variations in disease management and differences in 5-year survival rates between different NHL subtypes and countries were found. The majority of patients received first-line chemoimmunotherapy, with newer treatment options such as rituximab-based regimens showing improved clinical outcomes.
**Relevance**
The findings from our study are important for informing ongoing efforts and future strategies in improving NHL management in Latin America, highlighting potential differences in patient access to appropriate therapy between different regions.

In the past 15 years, there have been paradigm shifts in diagnosis, staging, and treatment of hematologic malignancies around the world. However, there is a paucity of real-world evidence (RWE) on NHL in Latin America, so it is unclear how these changes have been translated to clinical practice in the region.^2-4^ The WHO estimates that only 8% of Latin American populations are covered by cancer registries.^5^

The Hemato-Oncology Latin America Observational (HOLA) Registry study was conducted with the primary aim of quantifying demographic and clinical characteristics of patients who had diagnoses of multiple myeloma (MM), chronic lymphocytic leukemia (CLL), or NHL and received care at tertiary care hospitals. MM- and CLL-related epidemiology and treatment outcome data from the HOLA study have been published elsewhere.^6-8^ This study reports the treatment patterns and clinical outcomes of patients who were diagnosed with NHL and received systemic therapy.

## METHODS

### Study Design

This multicenter, observational study retrospectively reviewed medical records from January 1, 2006, to December 31, 2015, of adult Latin American patients diagnosed with MM, CLL, or NHL (NCT02559583) from 30 oncology hospitals in seven countries: Argentina (five), Brazil (nine), Chile (one), Colombia (five), Mexico (six), Panama (three), and Guatemala (one). Participating hospitals were selected based on their experience in providing clinical care for hematologic patients, geographic and practice type representativeness, and willingness to comply with study requirements. Data were collected via retrospective chart reviews conducted by trained medical abstractors using standardized data collection forms.

### Study Participants

An inception cohort of patients with NHL who satisfied the following criteria were included in the analysis: (1) incident or prevalent NHL diagnosis between January 1, 2006, and December 31, 2015; (2) patient age ≥ 18 years at the time of the first observed diagnosis; (3) 1 year or more of patient data after the first observed diagnosis (except in the event of patient death within 1 year of being diagnosed); and (4) patient (or legal representative) able and willing to provide informed consent (except in the event of obtaining a waiver of informed consent). Participants in clinical trials were excluded.

### Study Bias

Selection bias was minimized by applying broad eligibility criteria, recruiting patients from a diverse pool of clinical sites, and screening and identifying eligible patients consecutively. To minimize measurement bias associated with inaccurate assessments and variable content of medical records, chart abstractors underwent comprehensive central training in performing reviews, and clear definitions of variables of interest were provided to ensure accurate assessment of desired data elements. Missing values did not contribute to denominators used to estimate percentages.

### Statistical Methods

Demographics and clinical variables were characterized using descriptive statistics, including measures of central tendency (mean and median) and spread (variance, range, minimum, and maximum) for continuous variables (eg, age at diagnosis) and frequency distributions (No., %) for categorical variables (eg, sex). Outcome measures for overall survival (OS) and progression-free survival (PFS) were defined as summarized in Table 1. Patients who were lost to follow-up or alive at the last available data extraction were censored at the date of last consult. PFS was censored at the time of the last consult date available.

**TABLE 1:**
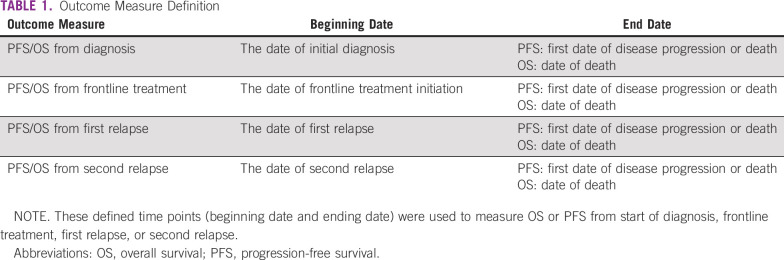
Outcome Measure Definition

OS and PFS were assessed following Kaplan-Meier methodology, with results described when the number of patients at risk at the beginning of the observation period was ≥ 10. Outcome analyses were conducted in four B-cell NHL subtypes: diffuse large B-cell lymphoma (DLBCL), follicular lymphoma (FL), mantle-cell lymphoma (MCL), and mucosa-associated lymphoid tissue (MALT) lymphoma but were not further analyzed by country. Because of the limitation of sample sizes, associations were only examined for different treatment regimens and age at diagnosis on OS and PFS for DLBCL and FL, estimated using the Cox proportional hazard model. Statistical analyses were conducted using SAS version 9.4 (SAS Institute, Cary, NC).

## RESULTS

### Overview of Patients With NHL Included in the Study

A total of 2,967 patients with NHL were included from Mexico (28.4%) Argentina (20.4%), Colombia (16.6%), Brazil (15.5%), Chile (15.3%), and Panama/Guatemala (3.8%). The median follow-up duration was 2.20 years (range < 0.1-11.8; n = 2,821). Overall, 691 (23.3%) patients died during follow-up and 1,571 (52.9%) remained under follow-up at the end of the study period.

Of these 2,967 patients, 2,948 had accurate NHL subtype information: 2,518 (85.4%) were classified as B-cell NHL including 1,457 (57.9%) DLBCL, 578 (23.0%) FL, 183 (7.3%) MCL, 90 (3.6%) CLL/small lymphocytic lymphoma, 84 (3.3%) MALT lymphoma, 60 (2.4%) Burkitt, 35 (1.4%) lymphoplasmacytic, and 31 (1.2%) B-lymphoblastic lymphoma. Of the remainder, 250 (8.5%) were diagnosed with T-cell NHL and 180 (6.1%) as other types of lymphoma (which may include subtypes of marginal zone lymphoma other than MALT; Table 2).

**TABLE 2:**
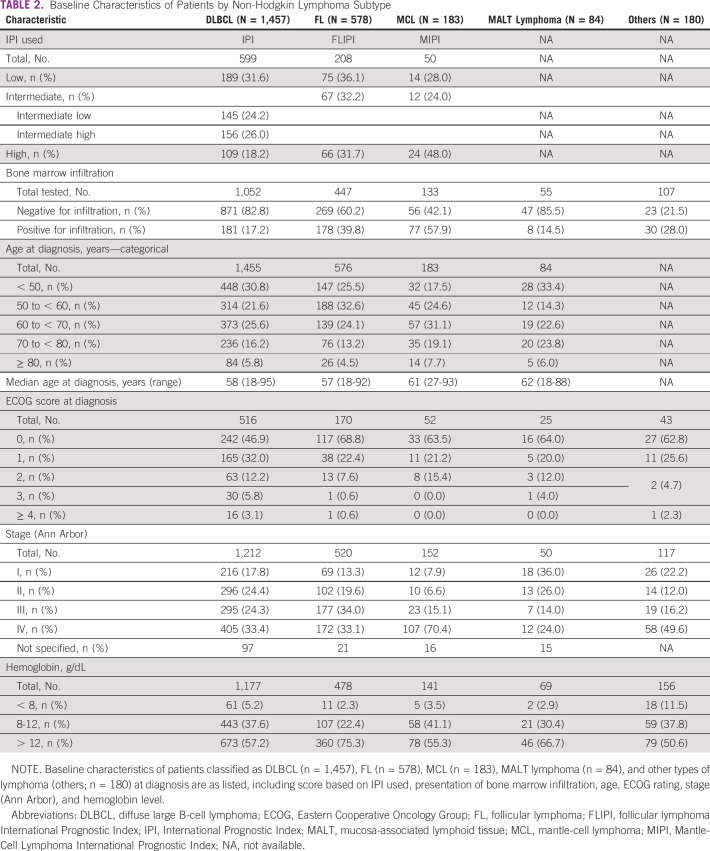
Baseline Characteristics of Patients by Non-Hodgkin Lymphoma Subtype

### Treatment Characteristics

Most patients with DLBCL received chemoimmunotherapy regimens (n = 1,420 of 1,457; 97.5%), of which the most commonly reported was rituximab, cyclophosphamide, doxorubicin, vincristine, and prednisone (R-CHOP; n = 998 of 1,420; 70.3%) and CHOP (excluding rituximab; n = 208 of 1,420; 14.6%). The median duration from initial diagnosis to treatment for DLBCL was 0.8 months (range 0-23.1; n = 1,198).

The majority of patients with FL received chemoimmunotherapy (n = 546 of 578, 94.5%). Approximately 70% received CHOP-like regimens combined with rituximab: R-CHOP (n = 280 of 546, 51.3%) or rituximab, cyclophosphamide, vincristine, and prednisone (RCVP; n = 96 of 546, 17.6%). A further 19.2% of patients received chemotherapy-only regimens, typically CHOP (n = 70 of 546, 12.8%) or cyclophosphamide, vincristine, and prednisone (CVP; n = 35 of 546, 6.4%). The median duration from initial diagnosis to treatment was 1.1 months (range 0-74.7; n = 467 of 578), which could be interpreted as the median watch and wait period.

For MCL, the majority of patients received chemotherapy (n = 172 of 183, 94.0%). Commonly prescribed regimens included R-CHOP (n = 71 of 172, 41.3%); CHOP (n = 28 of 172, 16.3%); hyper cyclophosphamide, vincristine, and doxorubicin (hyper CVAD; n = 18 of 172, 10.5%); and RCVP (n = 9 of 172, 5.2%). The median duration from initial diagnosis to treatment was 0.9 months (range 0-63.3; n = 130 of 183).

A similar pattern was observed among patients with MALT lymphoma, with 79.8% (n = 67 of 84) receiving a chemotherapy regimen (R-CHOP [n = 24 of 67, 35.8%]; CHOP [n = 12 of 67, 17.9%]; RCVP [n = 9 of 67, 13.4%]; CVP [n = 6 of 67, 9.0%]; and rituximab monotherapy [n = 6 of 67, 9.0%]). The median duration from initial diagnosis to treatment was 2.3 months (range < 0.1-42.1; n = 57 of 84), which could be interpreted as the median watch and wait period.

Relapse or progression since start of treatment was reported for 29% (n = 392 of 1,351) of patients with DLBCL, 30% (n = 157 of 524) with FL, 46.8% (n = 72 of 154) with MCL, and 24.2% (n = 15 of 62) with MALT lymphoma. The use of transplant was reported for 6.2% (n = 88 of 1,420) of patients with DLBCL, 5.7% (n = 31 of 546) with FL, 13.4% (n = 23 of 172) with MCL and none with MALT lymphoma. Among patients who received a transplant, most had an autologous transplant (97.7%, 93.5%, and 90.9% in DLBCL, FL, and MCL, respectively); however, data on the line of therapy were not available.

### OS from Diagnosis by NHL Subtypes

**FIG 1:**
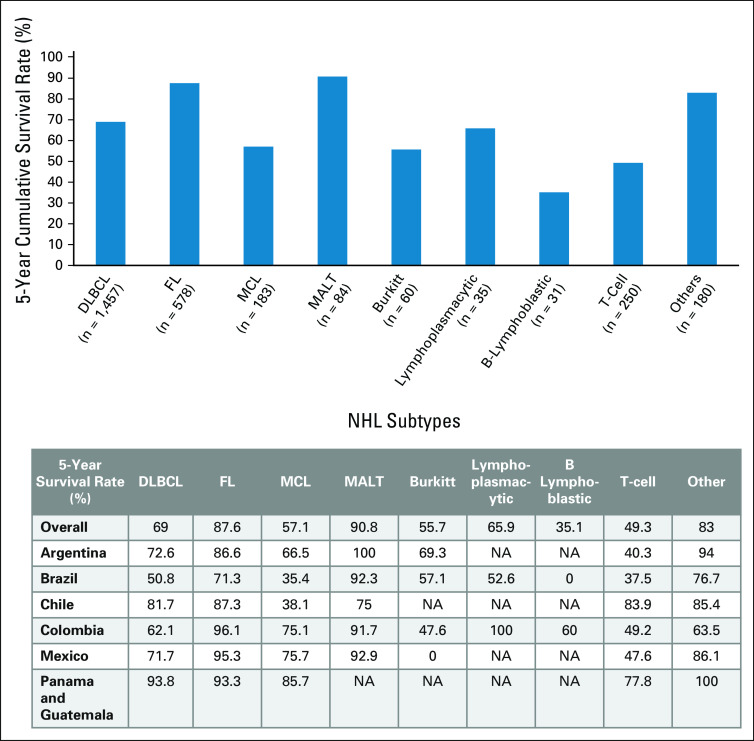
Five-year cumulative survival rate of patients by NHL subtypes from diagnosis. The 5-year cumulative survival rates of patients with DLBCL (n = 1,475), FL (n = 578), MCL (n = 183), MALT lymphoma (n = 84), Burkitt lymphoma (n = 60), lymphoplasmacytic lymphoma (n = 35), B-lymphoblastic lymphoma (n = 31), T-cell lymphoma (n = 250), and other types of lymphoma (others, n = 180) from diagnosis are illustrated in the top figure. The survival rate for each NHL subtype further stratified by country, expressed as percentage (%), are detailed in the bottom table. CLL, chronic lymphocytic leukemia; DLBCL, diffuse large B-cell lymphoma; FL, follicular lymphoma; MALT, mucosa-associated lymphoid tissue; MCL, mantle-cell lymphoma; NA, not available; NHL, non-Hodgkin lymphoma; SLL, small lymphocytic lymphoma.

Analysis of cumulative survival rates from diagnosis for NHL subtypes found that 5-year survival rates were highest for patients with MALT lymphoma (90.8%), followed by FL (87.6%), DLBCL (69.0%), and MCL (57.1%). Survival rates for each NHL subtype were further stratified by country (Fig 1).

Among patients for whom International Prognostic Index (IPI) was available at diagnosis (n = 599), the 5-year survival rate was highest in patients with low IPI (87.3%) and lowest in patients with high IPI scores (45.6%; Table 3).

**TABLE 3:**
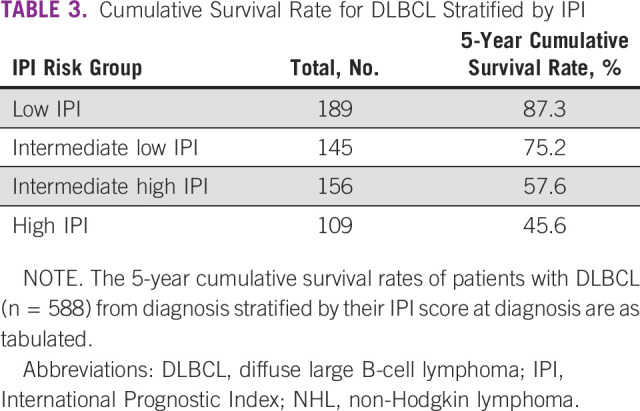
Cumulative Survival Rate for DLBCL Stratified by IPI

### OS/PFS From Start of Frontline Treatment

**FIG 2:**
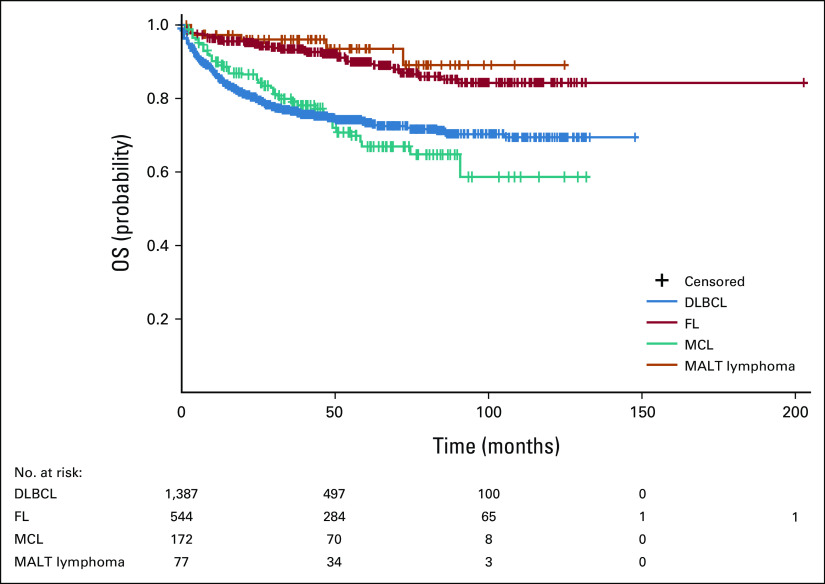
OS of patients by NHL subtypes from start of frontline treatment. Kaplan-Meier survival curves depicting the OS of patients with DLBCL (n = 1,387), FL (n = 544), MCL (n = 172), and MALT lymphoma (n = 77) from the start of first-line therapy; the results are described when the number of patients at risk at the beginning of the observation period was ≥ 10. DLBCL, diffuse large B-cell lymphoma; FL, follicular lymphoma; MALT, mucosa-associated lymphoid tissue; MCL, mantle-cell lymphoma; NHL, non-Hodgkin lymphoma; OS, overall survival.

Overall survival of patients with DLBCL, FL, MCL, and MALT lymphoma were analyzed from the start of frontline therapy (Fig 2). Patients with DLBCL age < 65 years at diagnosis were at lower risk of death compared with patients ≥ 65 years (hazard ratio [HR] = 0.732; *P* = .0161).

Of patients who experienced relapse or progression, the median time from treatment initiation to relapse/progression was 8.4 months (range < 0.1-92.6; n = 236) for those treated with R-CHOP, 7.6 months (range 0.7-57.6; n = 74) for CHOP, and 5 months (range 0.6-21.6; n = 14) for RCVP. For FL, patients treated with R-CHOP achieved a median time from treatment initiation to relapse/progression of 17.6 months (range < 0.1-74.4; n = 61), whereas patients treated with CHOP or RCVP achieved medians of 14.5 months (range 0.5-83.3; n = 29) and 9.8 months (range 0.1-70.6; n = 26), respectively.

For DLBCL, there were no statistically significant differences in risk of death between patients treated with CHOP versus R-CHOP (HR = 1.330; *P* = not significant) or RCVP versus R-CHOP (HR = 1.118; *P* = not significant). However, higher risk of death was observed for patients with FL treated with CHOP versus R-CHOP (HR = 2.508; *P* = .001), although no differences were seen for RCVP versus R-CHOP (HR = 1.066; *P* = not significant).

For patients with DLBCL, 5-year PFS rates for the three most common frontline regimens ranged from 44.9% to 56.2%, with a median PFS of 7.7 years (95% CI, 5.8 to not estimable (n.e.); n = 948) for patients receiving R-CHOP (Table 4). Similarly, 5-year PFS rates among patients with FL for the three most commonly used regimens were 42.5%-65.1%. There was no relationship between type of frontline regimen prescribed and age at diagnosis with PFS, for either DLBCL or FL.

**TABLE 4:**
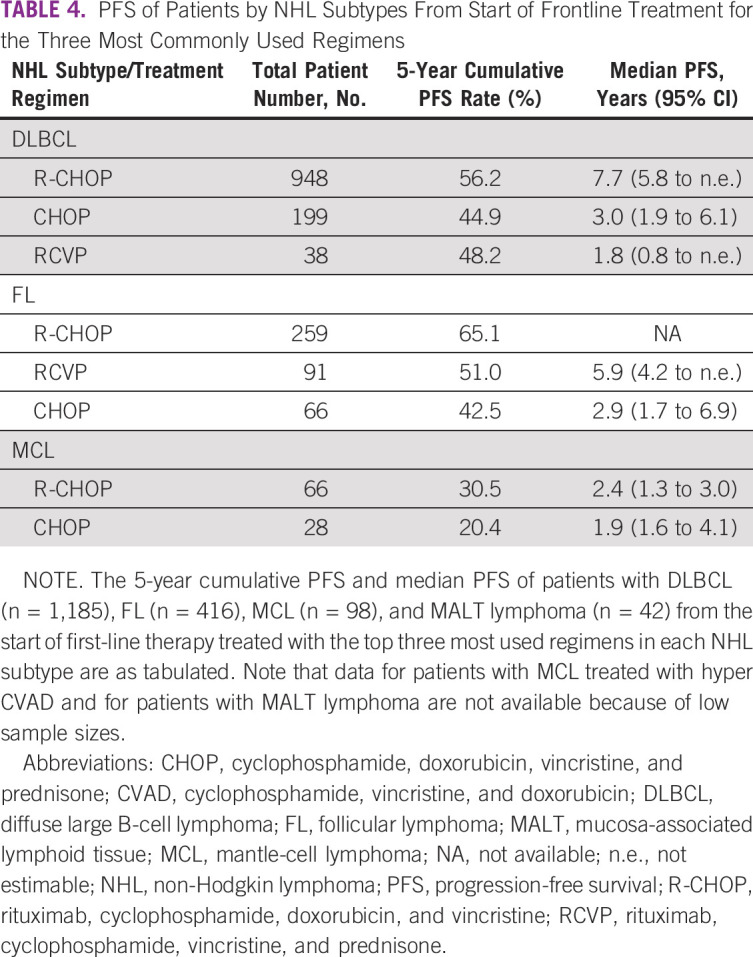
PFS of Patients by NHL Subtypes From Start of Frontline Treatment for the Three Most Commonly Used Regimens

### OS/PFS From First Relapse

**FIG 3:**
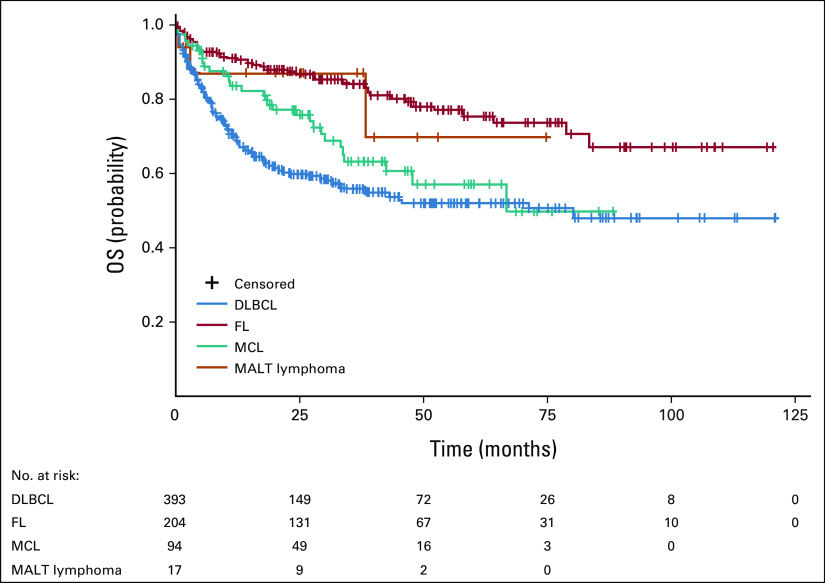
OS of patients by NHL subtypes from first relapse. Kaplan-Meier survival curves depicting the OS of patients with DLBCL (n = 393), FL (n = 204), MCL (n = 94), and MALT lymphoma (n = 17) from first relapse; the results are described when the number of patients at risk at the beginning of the observation period was ≥ 10. DLBCL, diffuse large B-cell lymphoma; FL, follicular lymphoma; MALT, mucosa-associated lymphoid tissue; MCL, mantle-cell lymphoma; NHL, non-Hodgkin lymphoma; OS, overall survival.

Overall survival of patients with DLBCL, FL, MCL, or MALT lymphoma from first relapse is shown in Figure 3. The median OS from first relapse was 6.6 years (95% CI, 3.1 to n.e.) for DLBCL, whereas median OS from first relapse was not reached for FL, MCL, or MALT lymphoma.

**FIG A1:**
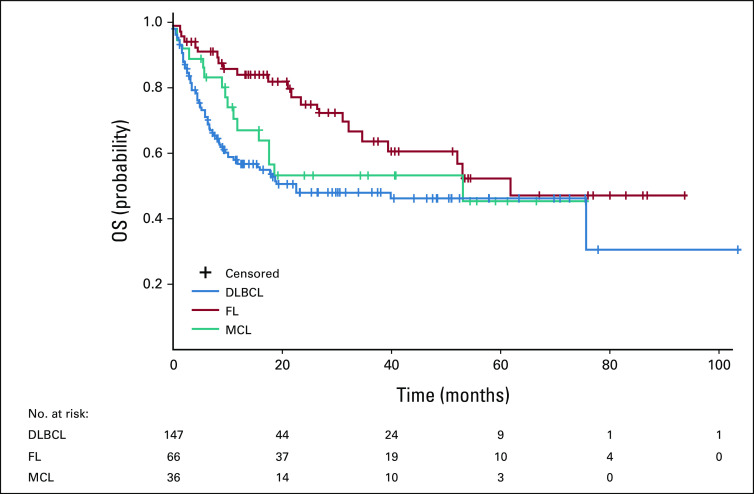
OS of patients by NHL subtypes from second relapse. Kaplan-Meier survival curves depicting the OS of patients with DLBCL (n = 147), FL (n = 66), and MCL (n = 36) from second relapse; the results are described when the number of patients at risk at the beginning of the observation period was ≥ 10. In total, 152 patients with DLBCL, 70 patients with FL, 38 patients with MCL, and 3 patients with MALT lymphoma received treatment for second relapse. For DLBCL, the median OS from second relapse was 1.9 years (95% CI, 0.43 to n.e.), whereas the median OS from second relapse for FL or MCL was 5.0 years (95% CI, 1.9 to n.e.) or 4.4 years (95% CI, 0.8 to n.e.), respectively. Outcome analysis was not conducted for MALT lymphoma because of small patient sample size. DLBCL, diffuse large B-cell lymphoma; FL, follicular lymphoma; MALT, mucosa-associated lymphoid tissue; MCL, mantle-cell lymphoma; n.e., not estimable; NHL, non-Hodgkin lymphoma; OS, overall survival.

**FIG A2:**
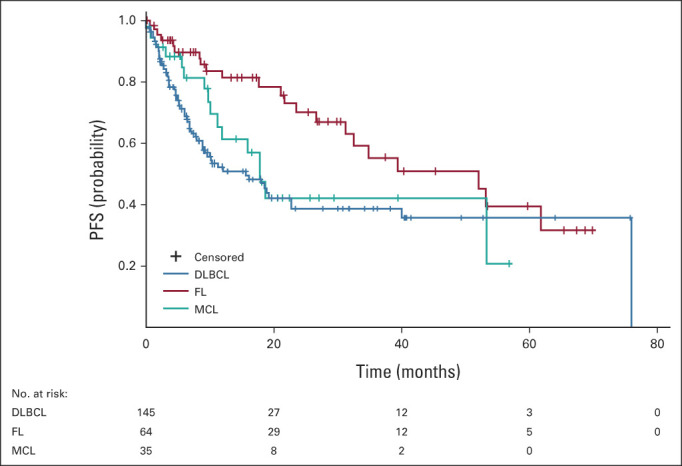
PFS of patients by NHL subtypes from second relapse. Kaplan-Meier survival curves depicting the PFS of patients with DLBCL (n = 145), FL (n = 64), and MCL (n = 35) from second relapse; the results are described when the number of patients at risk at the beginning of the observation period was ≥ 10. The median PFS from second relapse was 1.3 years (95% CI, 0.4 to 6.2) in DLBCL, 4.3 years (95% CI, 1.8 to n.e.) in FL, and 1.5 years (95% CI, 0.8 to 4.4) in MCL. DLBCL, diffuse large B-cell lymphoma; FL, follicular lymphoma; MCL, mantle-cell lymphoma; n.e., not estimable; NHL, non-Hodgkin lymphoma; PFS, progression-free survival.

The median PFS from first relapse was 2.3 years (95% CI, 0.4 to n.e.) for DLBCL and 2.6 years (95% CI, 1.1 to 3.9) for MCL. Median PFS was not reached in patients with FL or MALT lymphoma. Additional analyses on OS/PFS from second relapse are shown in Appendix Figs A1 and A2.

For DLBCL, the three most common treatments at first relapse were ifosfamide, carboplatin, and etoposide with/without rituximab (RICE/ICE; 21.1%, n = 86 of 407); etoposide, methylprednisolone, cytarabine, and cisplatin (10.1%, n = 41 of 207); and R-CHOP (8.8%, n = 36 of 407). Patients treated with RICE/ICE after first relapse were at significantly higher risk of death versus patients treated with R-CHOP (HR = 3.115; *P* = .0035). Similarly, the risk of disease progression after first relapse was also higher with RICE/ICE versus R-CHOP (HR = 3.846; *P* = .0011). However, we were not able to identify the first-line treatments prescribed to those patients who used R-CHOP as second-line rescue therapy.

## DISCUSSION

Management of patients with NHL has changed substantially over the past two decades with improved diagnostic techniques, newer treatment options, and consequently better treatment outcomes. However, RWE generation is necessary to better understand how these treatment improvements are implemented in individual countries. As RWE for NHL in Latin America is generally lacking,^2-4^ data presented here from the observational HOLA study provide a significant step in better understanding treatment pathways and outcomes for patients in the region. Of note are the cohort size of the patients with NHL from seven Latin American countries (n = 2,967) and the long-term 8-year follow-up period.

Among all NHL subtypes, DLBCL is the most common subtype reported globally (1,457, 57.9% in our study) and is typically treated using combination chemotherapy including CHOP and R-CHOP. In our study, the 5-year survival rates for DLBCL ranged from 45.6% in patients with high IPI to 87.3% in patients with low IPI. Improved outcomes were seen in our study when comparing R-CHOP versus CHOP, including 5-year PFS rates and median PFS; however, there was no statistical difference in risk of death or disease progression between the regimens. The reason for this is unclear, although may be due to the limitations of analyzing data from retrospective observational studies. Nonetheless, we found that patients with DLBCL treated with RICE/ICE after first relapse were at significantly higher risk of death and/or disease progression versus R-CHOP, thus signifying the importance of newer therapies in improving survival outcomes for patients.

FL accounts for around 20-30% of NHL cases in our study, with 51.3% of patients treated with chemotherapy receiving R-CHOP. Patients with FL achieved a 5-year overall survival rate of 87.6%, which was higher than observed for other NHL subtypes (except MALT lymphoma). These observations are in line with previously reported improvements in OS for patients with FL treated with rituximab-based regimens,^9,10^ as observed by comparing OS of patients diagnosed with FL during the rituximab era (1997-2003, median OS not reached) with earlier decades (median OS of 13.6-18.5 years).^9^ The results from our study also highlight the necessity of detecting FL earlier in Latin America as the median time from diagnosis to treatment was 1.1 months (range 0-74.7) for nearly 80% of the FL patient cohort in the HOLA study, who were mostly diagnosed at advanced stages of their disease and required systemic therapy.

MCL is an aggressive form of B-cell NHL. Relapsed MCL has been associated with poor responses and OS of < 3 years.^11^ Little has been reported on treatment outcomes of patients with MCL in Latin America, with one retrospective study conducted in Mexico identifying only 12 patients.^12^ The majority of patients with MCL in our study received chemotherapy, where 41.3% of patients received R-CHOP and 16.3% received CHOP. The 5-year survival rate for MCL was 57.1%, which compared poorly with rates for DLBCL and FL. The MCL cohort studied in the HOLA registry is smaller compared with other subtypes, as would be expected given that MCL is a rarer form of NHL. Despite this, our study provides useful insights into the management of the disease in this region, and greater international efforts should be considered to further understand outcomes of MCL in Latin America.

A cohort of 84 patients with MALT lymphoma were analyzed in our study. Given the indolent nature of the disease, specifically identifying the watch and wait subgroup proved challenging.^13^ However, the median time from diagnosis to treatment initiation reported for MALT lymphoma (2.3 months) could potentially be interpreted as such. Additionally, we were unable to categorize further subsets of marginal zone lymphoma lymphomas, such as splenic or extranodal subtypes, which may have been classified as others (n = 180).

Maintenance therapy is a known effective strategy in prolonging remission for patients with NHL after induction therapy or stem-cell transplantation. For example, the clinical value of rituximab maintenance in treatment of FL has been demonstrated in several real-world studies in the United States,^14^ Taiwan,^15^ and Czech Republic.^16^ Unfortunately, data on the use of maintenance therapy in low-grade lymphomas were not gathered in the HOLA study, with the exception of Argentina where rituximab maintenance was reported as the most common treatment for DLBCL at first or at second relapse (n = 11 of 74, 14.9% and n = 4 of 18, 22.2%, respectively).

There are certain limitations of this study, some of which are common to retrospective observational studies. First, missing data were observed for some variables because of limited information available in patient records. Second, although the total sample size was adequate, smaller sample sizes for some subgroups may have influenced findings, particularly those related to outcomes from Cox models. Third, PFS and OS analyses were affected by a large degree of loss to follow-up, especially from first and second relapse, limiting the precision of those analyses. PFS and OS analyses also assumed that patients with continued follow-up were representative of the original total patient cohort and may also be overly optimistic if, for example, an out-of-hospital death resulted in loss to follow-up but was not documented in the patient's medical chart. Collectively, caution should be exercised when interpreting our PFS and OS estimates, especially in comparison with other studies. Finally, censored and not censored patients may not have presented homogeneous characteristics at the beginning of the study.

Overall, this study summarizes the treatment outcomes for patients with NHL in Latin America and highlights some differences between participating countries which should be considered with care because of sample numbers and heterogeneity of patient characteristics. Nonetheless, disparities in survival outcomes between the seven participating countries (for example, survival rates for DLBCL, FL, and MCL were lowest in Brazil), potentially reflect regional differences in patient access to timely treatment. Socioeconomic, geographic, and cultural disparities in the region can affect outcomes for hematological malignancies, with some of the key challenges including limited numbers of hematologists and inadequate access to bone marrow transplant centers.^17^ Furthermore, although R-CHOP is the standard of care for DLBCL globally, nearly 15% of patients with DLBCL in our study were prescribed CHOP, which may be indicative of late adoption of novel treatments in this region.^18^ Furthermore, we found that management of second- and third-line patients was inconsistent, reflecting the lack of standard of care.

In conclusion, the HOLA study is the first large scale, real-world observational study to report treatment pathways and clinical outcomes for patients with NHL in multiple countries across Latin America. These data are important for informing local improvements in NHL management and for understanding how treatment practices differ compared with other regions in the world. Data gaps from this study highlight the real-world challenges of providing treatment and recording data across the region, indicating a need for further studies and support for treatment of NHL in Latin America.

## References

[1] Curado MP, de Souza DL: Cancer burden in Latin America and the Caribbean. Ann Glob Health 80:370-377, 2014 25512152 10.1016/j.aogh.2014.09.009

[2] Diumenjo MC, Abriata G, Forman D, et al: The burden of non-Hodgkin lymphoma in Central and South America. Cancer Epidemiol 44:S168-S177, 2016 (suppl 1) 27678319 10.1016/j.canep.2016.05.008

[3] Ortega V, Verastegui E, Flores G, et al: Non-Hodgkin's lymphomas in Mexico. A clinicopathological and molecular analysis. Leuk Lymphoma 31:575-582, 1998 9922048 10.3109/10428199809057617

[4] Muller AM, Ihorst G, Mertelsmann R, et al: Epidemiology of non-Hodgkin's lymphoma (NHL): Trends, geographic distribution, and etiology. Ann Hematol 84:1-12, 2005 15480663 10.1007/s00277-004-0939-7

[5] Bray F, Colombet M, Mery L, et al: Cancer Incidence in Five Continents, Vol. XI. IARC Scientific Publication No. 166. Lyon: International Agency for Research on Cancer. https://publications.iarc.fr/597

[6] Chiattone C, Gomez-Almaguer D, Pavlovsky C, et al: Results from Hemato-Oncology Latin America Observational Registry (HOLA) providing real world outcomes for the treatment of patients with CLL. Blood 128:5578, 2016

[7] Tietsche De Moraes Hungria V, Chiattone C, Pavlovsky M, et al: Epidemiology of hematologic malignancies in real-world settings: Findings from the Hemato-Oncology Latin America Observational Registry study. JCO Glob Oncol 5:1-19, 2019 10.1200/JGO.19.00025 PMC6882510 31774711

[8] de Moraes Hungria VT, Martínez-Baños DM, Peñafiel CR, et al: Multiple myeloma treatment patterns and clinical outcomes in the Latin America Haemato-Oncology (HOLA) Observational Study, 2008–2016. Br J Haematol 188:383-393, 2020 31392724 10.1111/bjh.16124 PMC7003731

[9] Tan D, Horning SJ, Hoppe RT, et al: Improvements in observed and relative survival in follicular grade 1-2 lymphoma during 4 decades: The Stanford University experience. Blood 122:981-987, 2013 23777769 10.1182/blood-2013-03-491514 PMC3739040

[10] Provencio M, Sabin P, Gomez-Codina J, et al: Impact of treatment in long-term survival patients with follicular lymphoma: A Spanish Lymphoma Oncology Group Registry. PLoS One 12:e0177204, 2017 28493986 10.1371/journal.pone.0177204 PMC5426713

[11] Schieber M, Gordon LI, Karmali R: Current overview and treatment of mantle cell lymphoma. F1000Res 7:F1000 Faculty Rev-1136, 2018 10.12688/f1000research.14122.1 PMC6069726 30109020

[12] Córdova-Ramírez AC, Sánchez-Valledor LF, Colón-Otero G, et al: Mantle cell lymphoma may have a different clinical course in Mexican Mestizos: Real-world data from a single center. Rev Invest Clin 73(2):94-9, 2021 10.24875/RIC.20000381 33075042

[13] Raderer M, Kiesewetter B, Ferreri AJM: Clinicopathologic characteristics and treatment of marginal zone lymphoma of mucosa-associated lymphoid tissue (MALT lymphoma). CA Cancer J Clin 66:152-171, 2016 10.3322/caac.21330 26773441

[14] Nastoupil LJ, Sinha R, Byrtek M, et al: The use and effectiveness of rituximab maintenance in patients with follicular lymphoma diagnosed between 2004 and 2007 in the United States. Cancer 120:1830-1837, 2014 24668580 10.1002/cncr.28659 PMC4265986

[15] Huang H-H, Wen Y-C, Chen H-M, et al: Rituximab maintenance improves overall survival in follicular lymphoma: A retrospective nationwide real-world analysis from Taiwan Cancer Registry Database. Cancer Med 7:3582-3591, 2018 30009424 10.1002/cam4.1622 PMC6089160

[16] Belada D, Prochazka V, Janikova A, et al: The influence of maintenance therapy of rituximab on the survival of elderly patients with follicular lymphoma. A retrospective analysis from the database of the Czech Lymphoma Study Group. Leuk Res 73:29-38, 2018 30195062 10.1016/j.leukres.2018.08.019

[17] Goss PE, Lee BL, Badovinac-Crnjevic T, et al: Planning cancer control in Latin America and the Caribbean. Lancet Oncol 14:391-436, 2013 23628188 10.1016/S1470-2045(13)70048-2

[18] Tilly H, Gomes da Silva M, Vitolo U, et al: Diffuse large B-cell lymphoma (DLBCL): ESMO clinical practice guidelines for diagnosis, treatment and follow-up. Ann Oncol 26:v116-v125, 2015 (suppl 5) 26314773 10.1093/annonc/mdv304

